# A Narrative Review on the Efficacy and Safety of Loop Diuretics in Patients With Heart Failure With Reduced Ejection Fraction and Preserved Ejection Fraction

**DOI:** 10.7759/cureus.45794

**Published:** 2023-09-22

**Authors:** Ruth Pius, God-dowell O Odukudu, Israel Olorundare, Deborah I Makanjuola, Rosemary Komolafe, Chidimma Njoku, Ogheneakpobor E Ubogun, Ramatu Muhammad, Elsie O Osiogo, Caleb Anulaobi

**Affiliations:** 1 Internal Medicine, Lincoln Medical Centre, Bronx, USA; 2 Internal Medicine, Delta State University, Abraka, NGA; 3 Surgery, Johns Hopkins University School of Medicine, Baltimore, USA; 4 Public Health, University of Alabama at Birmingham, Birmingham, USA; 5 Clinical Sciences, University of Ilorin, Ilorin, NGA; 6 Internal Medicine, Gomel State Medical University, Gomel, BLR; 7 Internal Medicine, Delta State University Teaching Hospital, Oghara, NGA; 8 Public Health, Johns Hopkins Bloomberg School of Public Health, Baltimore, USA; 9 Internal Medicine, Ahmadu Bello University Teaching Hospital, Zaria, NGA; 10 Internal Medicine/Infectious Diseases, Ekiti State Ministry of Health and Human Services, Ado-Ekiti, NGA; 11 Medicine and Surgery, Abia State University, Uturu, NGA

**Keywords:** hfpef, hfref, safety, efficacy, loop diuretics

## Abstract

To date, loop diuretics are the mainstay treatment for decongestion in patients with acute decompensated heart failure (HF). In clinical practice, loop diuretics have also been utilized for patients with chronic HF with reduced and preserved ejection fractions. There is a paucity of quality evidence of the effect of loop diuretics use and dosing on clinical outcomes in HF patients beyond symptomatic relief. In this review, we aimed to summarize recently published data on the use of loop diuretics in patients with HF, focusing on efficacy and safety outcomes in patients with HF with reduced and preserved ejection fraction. We searched EMBASE, PubMed, CINAHL, and the “Web of Science” databases. Cohort studies and randomized controlled trials published after 2018 and written in English were included in this review. Case reports, case series, cross-sectional studies, review articles, commentaries, articles published more than five years ago, and studies involving children were excluded. Results were divided into the efficacy and safety of loop diuretics in HF with reduced ejection fraction (HFrEF) and HF with preserved ejection fraction (HFpEF). A registry-based study included in our review observed a reduced 30-day all-cause mortality in patients with HFrEF receiving loop diuretics compared to those not receiving loop diuretics (HR=0.73; 95% CI=0.57-0.94; p=0.016), but there was no statistically significant association at the 60-day follow-up of the same group of patients. Most studies reviewed showed that the choice of loop diuretics did not influence clinical outcomes such as mortality and HF rehospitalization in patients with HF with reduced and preserved ejection fraction despite differences in oral bioavailability and half-life. Studies have consistently shown that patients with HF who receive a higher dose of loop diuretics are likely to experience a decline in renal function and hypotension, regardless of their type of HF. Discontinuation or reduction of the dose of loop diuretics should be considered in patients with HF after decongestion.

## Introduction and background

Heart failure (HF) significantly contributes to morbidity and mortality, with about 64.3 million people suffering from it worldwide [1,2]. According to the American Heart Association, about 6.2 million adults aged ≥20 years had HF between 2013 and 2016 in the US, and the prevalence is said to be on the rise with an increasing aging population [3]. It is a complex clinical syndrome characterized by inadequate ventricular filling or reduced ejection fraction due to a functional or structural cardiac condition [4]. Common clinical features of HF include dyspnea, orthopnea, easy fatigability, edema, and poor exercise tolerance. Approximately 50% of patients with HF are estimated to suffer from HF with reduced ejection fraction (HFrEF), whereas the remaining 50% have a normal or near-normal ejection fraction [5]. Although mortality rates are similar for both HFrEF and HF with preserved ejection fraction (HFpEF) [5], studies have demonstrated that they exhibit distinct hemodynamic traits and respond differently to treatment [6].

The pathophysiology of HF involves the activation of the renin-angiotensin-aldosterone system (RAAS), leading to the retention of sodium and water [7]. To date, loop diuretics are the mainstay treatment for decongestion in patients with acute decompensated HF [7,8]. Loop diuretics are also commonly used in clinical practice for patients with chronic HF with reduced and preserved ejection fractions. They have been shown to improve the quality of life of patients with heart by relieving dyspneic symptoms and, as a result, increasing functional capacity [9].

Despite the widespread use of loop diuretics for HF, quality evidence of the effect of loop diuretics on outcomes beyond symptomatic relief is scarce. Some recent data from randomized controlled trials, such as the Heart Failure: A Controlled Trial Investigating Outcomes of Exercise Training (HF-ACTION) trial and the Diuretic Optimization Strategy Evaluation (DOSE) trial, have provided new insights on the safety and efficacy of loop diuretics in various categories of patients with HF [10,11].

According to the 2022 AHA/ACC/HFSA Guideline for the Management of Heart Failure, it is recommended to use the lowest effective dose of loop diuretics that can maintain a euvolemic state while reducing congestion in patients. However, there are no particular recommendations regarding the loop diuretic choice [1]. Torsemide and bumetanide, which are not as commonly used as furosemide, have better oral bioavailability but no difference in clinical outcomes compared to furosemide [12].

Although a high dose of loop diuretics is often used to decongest patients with HF, they have been shown to have adverse effects, such as worsening renal function and electrolyte imbalance [13]. For instance, the comparison of the response to loop diuretics of patients with acute decompensation of HFrEF and HFpEF in the Diuretic Optimization Strategy Evaluation (DOSE) trial showed that patients with HFpEF are at greater risk of hypotension and worsening renal function due to a more significant decrease in plasma volume when treated with high dose loop diuretics [11]. This review article seeks to summarize recently published data on the use of loop diuretics in patients with HF, focusing on efficacy and safety outcomes in HFrEF and HFpEF.

Classification of HF

HF is often classified using the left ventricular ejection fraction (LVEF). LVEF affects the choice of treatment and prognosis for HF. Ejection fraction is often a criterion for patient selection in many HF studies. The 2022 AHA/ACC/HFSA guideline for the management of HF categorizes HF based on LVEF into (1) HFrEF with LVEF ≤40%; (2) HF with improved ejection fraction (HFimpEF), which is considered to be a subgroup of HFrEF, with previous LVEF ≤40% and follow-up measurement of LVEF >40%; (3) HF with mildly reduced ejection fraction (HFmrEF), with LVEF of 41%-49%, plus evidence of spontaneous or provokable increased LV filling pressures; and (4) HFpEF with LVEF ≥50%, plus evidence of spontaneous or provokable increased LV filling pressures [1].

Despite this classification, various studies have used several cutoff points to categorize patients with HF. For instance, HFrEF has been classified as LVEF of <35% or <40%, while HFpEF is often classified as EF >40%, >45%, or 50% [1].

Pathophysiology of HFrEF

HFrEF occurs due to systolic dysfunction precipitated by conditions that cause cardiac myocyte injury and death. The heart's pumping capacity and ejection fraction are reduced due to harmful stimuli, such as ischemia, volume overload, hypertension, toxins, arrhythmia, and genetic defects in myocyte contractile proteins, among others ​[14]. Various compensatory mechanisms are activated to maintain systemic perfusion when left ventricular dysfunction occurs. These mechanisms include the RAAS, the sympathetic nervous system, and inflammatory cytokines. The activation of these systems allows for an improvement in cardiac function and systemic perfusion due to cardiac remodeling, peripheral vasoconstriction, and increased end-diastolic volumes. However, the beneficial effects of these changes to cardiac function and structure are transient and have long-term harmful effects that worsen cardiac function ​[15]. HFrEF is more common in males, and its etiology includes coronary artery disease, dilated cardiomyopathy, valvular heart disease, hypertension, arrhythmias, infiltrative disorders, genetic, toxin-mediated, chronic obstructive pulmonary disease, etc. [1].

Pathophysiology of HFpEF

HFpEF is HF accompanied by evidence of spontaneous or provokable increased LV filling pressures ​[1]. Mechanisms that may drive HFpEF include diastolic dysfunction, left heart congestion, left atrial hypertension, and systemic microvascular inflammation. In HFpEF, the systolic function is adequate [16,17]. When there is diastolic dysfunction, it leads to an increase in the left ventricular filling pressures. Additionally, the left ventricular wall thickens, and the size of the ventricular cavity decreases [18,19]. Elevated left ventricular filling pressures promote vascular remodeling over time, which greatly impacts the pulmonary veins and capillaries and may lead to pulmonary hypertension. The ventricular wall stiffness, often seen in HFpEF, causes slow LV relaxation, reducing end-diastolic volume and stroke volume, especially during periods of exercise [18]. ​Our understanding of the pathophysiology of HFpEF remains limited and continues to improve with new data from clinical trials [20]. HFpEF is common in elderly females​ and is often linked with other health conditions, such as chronic kidney disease, obesity, arterial hypertension, atrial fibrillation, and diabetes mellitus.

Loop diuretics

Loop diuretics are a group of highly potent diuretics. They are also known as high-ceiling diuretics. They have a vast role in clinical medicine, including managing and treating hypertension, acute pulmonary edema, severe hypercalcemia, and fluid overload in conditions such as HF, nephrotic syndrome, and cirrhosis.

Pharmacodynamics and pharmacokinetics of loop diuretics

Loop diuretics function at the thick ascending limb of the renal tubule and inhibit the Na-K-2Cl transporter [21]. Examples include furosemide, torsemide, azosemide, bumetanide, and ethacrynic acid [5,21]. They can be administered orally or parenterally [21]. Torsemide has a longer duration of action and better oral bioavailability than furosemide [12] (Table 1). Loop diuretics are organic anions and tightly bind to plasma proteins; hence, their filtration into the renal tubules is limited. However, the organic acid transport system in the proximal tubule effectively secretes them [22]. While furosemide is mainly excreted unchanged, torsemide and bumetanide are eliminated by the renal and hepatic routes.

**Table 1:** Oral bioavailability and half-life of loop diuretics

Loop diuretic	Oral bioavailability	Half-life (hours)
Torsemide	80%	3-4
Furosemide	50%	1.5-2
Bumetanide	80%	1
Ethacrynic acid	100%	1
Azosemide	12%	2-3

Adverse effects of loop diuretics

Adverse effects of loop diuretics include hyponatremia, hypochloremia, hypomagnesemia, hypokalemia, metabolic alkalosis, dehydration, hypertriglyceridemia, postural hypotension, and syncope, among others [21,23].

Role of loop diuretics in the management of HFrEF and HFpEF

According to the 2022 AHA/ACC/HFSA Guideline for the Management of HF, loop diuretics are the preferred diuretic agents for most patients with HF [1]. Loop diuretics are used to decongest patients with HFrEF with fluid overload/retention and should be combined with other guideline-directed medical therapy (GDMT). GDMT is the gold standard for managing HFrEF. GDMT involves starting specific medications that have been proven to decrease mortality in HFrEF [1]. These include renin-angiotensin system inhibitors (ACEIs, ARBs, and ARNIs), beta-blockers, mineralocorticoid receptor antagonists, and sodium-glucose cotransporter (SGLT) 2 inhibitors. Intravenous loop diuretics are commonly given to patients with HFrEF while hospitalized, and oral formulations are continued at discharge for those with persistent congestion.

Loop diuretics application in HFpEF has similar clinical indications as in HFrEF [1]. However, achieving euvolemia in patients with HFpEF is a delicate process, as elevated left ventricular filling pressures cause a reduction in preload, and excessive diuresis may lead to a further reduction in cardiac output, hypotension, and decreased renal function [24,25]. Hence, largely reduced doses of loop diuretics may be used in most cases of HFpEF compared to HFrEF [24].

## Review

Methodology

Research Strategy

We searched four databases: EMBASE (Excerpta Medica database), PubMed, CINAHL, and “Web of Science” using specific search terms. Search terms were “Loop diuretic,” “Heart failure,” and ‘‘Ejection fraction." We searched for recent articles using a five-year limit; articles written after 2018 were included for further review.

Inclusion Criteria

Original articles written in English after 2018, cohort, or controlled trials involving adults aged >18 years were included. For this review, we carefully chose studies that reported the efficacy and safety of loop diuretics when used to manage HF in patients.

Exclusion Criteria

Case reports, case series, cross-sectional studies, review articles, commentaries, and articles published more than five years ago were excluded, as well as articles not written in English.

Results

**Figure 1:**
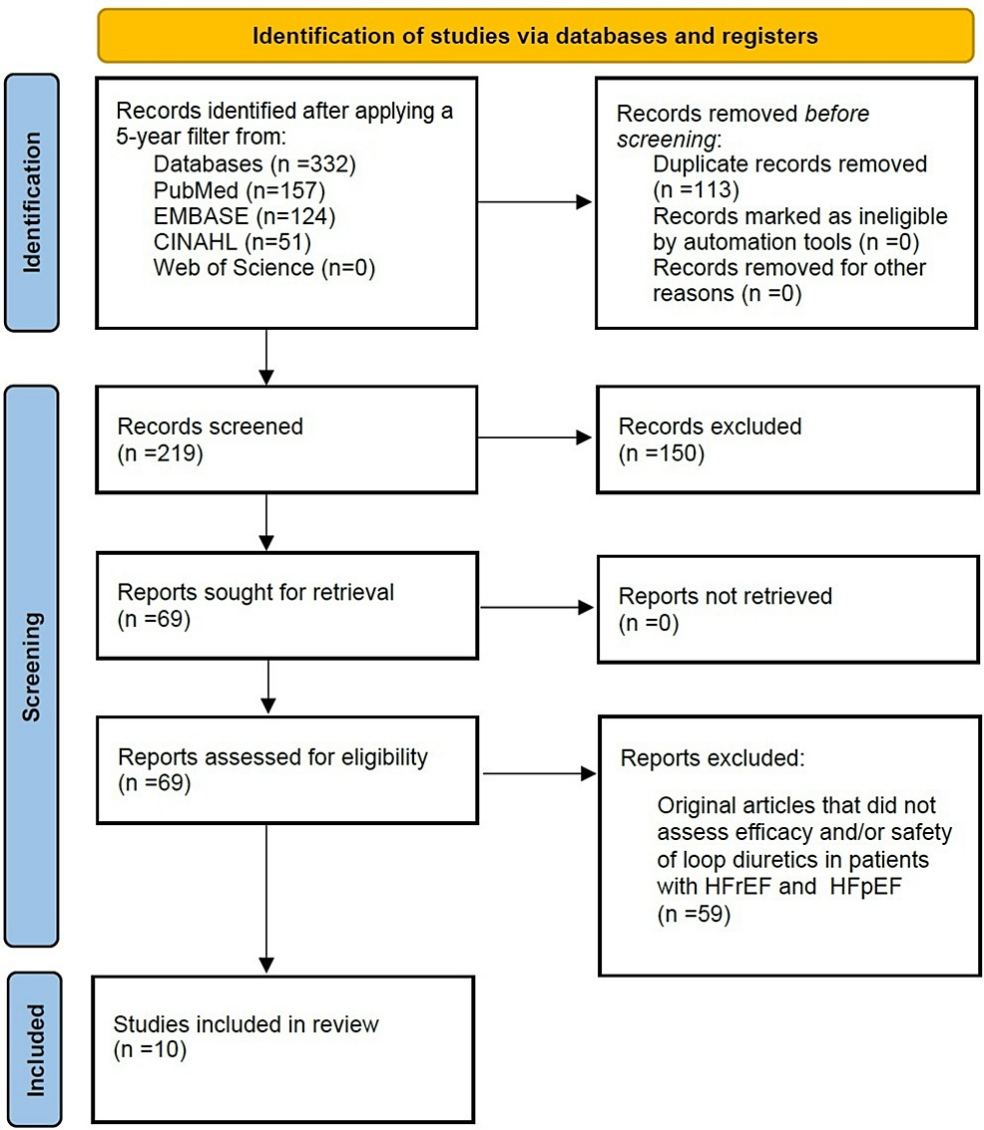
Flow diagram showing the selection process of the included articles used in this review

Our data search yielded 332 articles. Removing duplicates left 219 articles, and these were screened using their titles and abstracts. A secondary review was then carried out by reading the full text of the articles and using the inclusion and exclusion criteria to eliminate articles not possessing the required data for this review. This resulted in the selection of 10 articles that were relevant to our objective (Figure 1). A summary of the included articles' characteristics and findings is presented in Table 2.

**Table 2:** Characteristics and summary of findings of the articles included in this review HF: Heart Failure; LVD: Left Ventricular Dysfunction; LVEF: Left Ventricular Ejection Fraction; SNS: Sympathetic Nervous System; HFrEF: Heart Failure with Reduced Ejection Fraction; HFpEF: Heart Failure with Preserved Ejection Fraction; ARB: Angiotensin Receptor Blocker; LD: Loop Diuretics

S/N	Author/Year	Study Design	Study Population /Sample Size	Efficacy and Safety Parameters	Summary	Limitation of Study
1.	Täger et al., 2019 [26]	A retrospective observational study.	6,293 patients with chronic HF using either bumetanide, furosemide, or torsemide were identified in three European HF registries.	All-cause mortality. The study aimed to investigate whether the choice of individual loop diuretics (bumetanide, furosemide, or torsemide) has differential effects on survival in patients with chronic HF.	Bumetanide and furosemide were associated with higher mortality compared to torsemide treatment. However, when patients were individually matched based on propensity scores for receipt of the individual drug and dose-equivalents, there was no significant association between loop diuretic choice and all-cause mortality in any of the matched samples.	As an observational study, there could be potential confounding factors that may influence the results. The study design does not allow for establishing a cause-and-effect relationship between loop diuretic choice and mortality outcomes. The study focused on patients from three European HF registries, which may limit the generalizability of the findings to other populations or regions. The study might not have captured all potential variables that could impact mortality in patients with chronic HF.
2.	Mentz et al., 2023 [27]	An open-label, pragmatic, randomized trial.	2,859 participants hospitalized with heart failure (regardless of ejection fraction) at 60 hospitals in the United States.	All-cause mortality or all-cause hospitalization, and total hospitalizations.	The study found no significant difference in all-cause mortality between the torsemide and furosemide groups over 12 months following randomization. Both groups had similar rates of all-cause mortality. The results were consistent across various patient subgroups based on ejection fraction status.	The study has certain limitations, including loss of follow-up, patient crossover, and non-adherence. A total of 113 patients withdrew consent from the trial before completion. These factors may have impacted the interpretation of the findings. The pragmatic design of the trial also means that dosages of the loop diuretics were investigator-selected, potentially introducing variability in treatment. Additionally, the study's follow-up period was limited to 12 months for specific outcomes, which may not capture the longer-term effects of the interventions.
3.	Imaeda et al., 2022 [28]	The study was based on data from a prospective multicenter registry.	2,680 patients.	Hospital readmissions, all-cause death.	Patients in the long-acting LD group had a lower risk of the composite outcome (all-cause death or HF re-admission) than the short-acting LD group. Long-acting loop diuretics were associated with a lower risk of all-cause death and HF re-admission than short-acting loop diuretics. Subgroup analyses showed that using long-acting LD was associated with favorable outcomes, mainly in younger patients with reduced ejection fraction.	As the study is observational, it can only establish associations and cannot prove causation. The study used data from a registry, which might have inherent limitations, such as potential missing data or selection bias. The study focused on patients from a specific region (West Tokyo), which may limit the generalizability of the findings to other populations or regions.
4.	Faselis et al., 2020 [29]	Observational cohort study.	25,345 patients.	30-day all-cause mortality, 30-day heart failure readmission, and 30-day all-cause readmission.	The study found that older patients who were not taking diuretics before hospitalization for HF decompensation and received a discharge prescription for loop diuretics had significantly better 30-day clinical outcomes than those not discharged on loop diuretics. The loop diuretic group had a lower risk of 30-day all-cause mortality and 30-day HF readmission than those not using loop diuretics. However, these associations were not statistically significant during 60 days of follow-up.	As an observational cohort study, this study has inherent limitations, including potential selection bias and the inability to establish causal relationships between loop diuretic use and clinical outcomes. Observed significant associations may be sensitive to unmeasured confounding factors. Additionally, the study relied on registry data, which may have limited information on specific variables or outcomes. The findings of this study may only be generalizable to some populations, as the study specifically focused on older patients with HF.
5.	Ter Maaten et al., 2020 [30]	A retrospective observational study.	2,338 patients.	HF Hospitalization, all-cause mortality, Worsening renal function.	Higher doses of loop diuretics were associated with adverse clinical outcomes.	As the study is observational, it can only establish associations and cannot prove causation. They were also unable to exclude residual confounding, after propensity matching.
6.	Ruocco et al., 2019 [31]	Randomized controlled trial (RCT).	121 patients.	The cardiovascular (CV) death/heart failure (HF) re-hospitalization rate and daily weight loss per 40 mg of furosemide.	The study found that patients in the high-dose group of intravenous loop diuretic administration had a significantly higher rate of cardiovascular death or heart failure re-hospitalization compared to the low-dose group. The low diuretic response measured during the entire intravenous administration period better predicted poor prognosis than the diuretic response measured in the early phases. Both low diuretic response and high intravenous diuretic dose were related to poor prognosis.	As a post-hoc analysis of an existing trial, this study may be subject to selection bias and other limitations inherent to retrospective analyses. The diuretic dose regimen could partially influence the results in this study during the first 12 hours before randomization.
7.	Onitsuka et al., 2019 [32]	Prospective observational study.	137 patients.	Cardiac events (cardiac death or re-hospitalization due to the deterioration of HF).	The study aimed to investigate the relationship between short-acting loop diuretic (furosemide) doses and outcomes in patients with LVD and reduced LVEF. Patients receiving high-dose short-acting loop diuretics (≥40 mg/day of furosemide) had a higher risk of cardiac events than those receiving lower doses.	As an observational study, there is a potential for confounding factors that could influence the results. The sample size of one hundred thirty-seven patients might be relatively small, which may limit the generalizability of the findings. The study focused on short-term outcomes, so the long-term effects of high-dose furosemide and cardiac SNS abnormalities on outcomes need further investigation.
8.	He et al., 2021 [33]	A prospective, randomized, double‐blind, controlled trial.	300 patients.	Freedom from congestion at 72 hours, weight change, worsening renal function, and change in creatinine and cystatin C at 72 hours.	The study found that acute heart failure patients with HFrEF and HFpEF responded differently to aggressive diuresis. In HFrEF patients, aggressive diuresis with high-dose furosemide resulted in more favorable outcomes, including more significant fluid and weight loss, reduced congestion, and lower risk of adverse events. In HFpEF patients. Aggressive diuresis with high-dose furosemide led to a significant increase in creatinine and cystatin C levels without significant improvement in fluid and weight loss or risk reduction.	The sample size was limited, so the composite clinical outcome findings should be validated in a larger data set. The HFpEF subgroup of patients in this study includes both HFpEF and heart failure with mid‐range ejection fraction patients according to the latest guidelines.
9.	Parajuli et al., 2020 [34]	A retrospective single-center study.	445 patients.	30-day hospital readmission rate.	The overall 30-day readmission rate in the HFpEF cohort was 29%. Loop diuretics alone and combined with ARBs or beta-blockers were associated with a lower risk of 30-day hospital readmission. However, on multivariate logistic regression analysis, only loop diuretics were independently associated with a lower risk of hospital readmission in patients with HFpEF.	This was a retrospective study, as such the design is susceptible to inherent limitations, including selection bias and reliance on the available data. The study was conducted at a single center, which might limit the generalizability of the findings to other healthcare settings or populations. The study does not detail other patient characteristics or comorbidities that could influence readmission rates. Additionally, the study's findings are based on a specific time frame (30-day readmission), and longer-term effects of medication combinations were not evaluated.
10.	Sharma et al., 2018 [35]	A prospective randomized clinical trial.	90 patients.	All-cause mortality, 30-day HF readmission rate, change in 6-min walk distance at 72 hours, worsening renal function.	The study aimed to determine the best diuretic treatment strategy for hospitalized patients with HFpEF. Continuous infusion of furosemide was found to be associated with a higher increase in creatinine and a greater risk of WRF compared to intermittent bolus.	Potential limitations may include the relatively small sample size and the single-center design, which may limit the generalizability of the findings. Additionally, the study's duration and follow-up period might not be sufficient to capture long-term outcomes.

Efficacy of loop diuretics in HFrEF

All-Cause Mortality and HF Re-hospitalization Rate

Faselis et al., in their analysis of patients hospitalized for HF in the Medicare-linked OPTIMIZE-HF registry, observed a reduced 30-day all-cause mortality in patients with HFrEF receiving loop diuretics compared to those not receiving loop diuretics (HR=0.73, 95% CI=0.57-0.94, p=0.016). Additionally, they noted a significantly lower risk of 30-day HF readmission in the group of patients with HFrEF receiving loop diuretics (HR=0.79, 95% CI=0.63-0.99, p=0.037) [29]. This effect was not seen at the 60-day follow-up of the same group of patients [29]. The reduction in 30-day all-cause mortality may be attributable to the alleviation of fluid overload symptoms and a decreased risk of readmission.

Täger et al. reported no significant association between the loop diuretic used and all-cause mortality in any of their matched samples (bumetanide versus furosemide, HR=1.03, 95% CI=0.93-1.14, p=0.53; bumetanide versus torsemide, HR=0.98, 95% CI=0.78-1.24, p=0.89; furosemide vs. torsemide, HR=1.02, 95% CI=0.84-1.24, p=0.82) from their propensity score-matched analysis of chronic HF patients from three European HF registries [26]. The same outcomes were observed when analyzing subgroups of patients with left ventricular ejection fraction equal to or less than 35% compared to those with greater than 35% [26]. Similarly, Mentz et al. reported no significant difference in all-cause mortality among patients with HF with EF <40% taking furosemide compared to torsemide for 12 months post-hospitalization (HR=1.14, 95% CI=0.94-1.37) [27]. Imaeda et al. reported that the use of long-acting loop diuretics such as torsemide was significantly linked with a reduced risk of composite outcome (HR=0.58, 95% CI=0.42-0.82, p=0.002), including all-cause death (HR=0.51, 95% CI=0.30-0.85, p=0.010) and HF re-admission (HR=0.67, 95% CI=0.46-0.98, p=0.038) compared to short-acting loop diuretics in patients with HFrEF [28]. Compared to the other two studies, in Imaeda et al.'s work, the median follow-up period was more extended, at 2.1 years. It is suggested that this effect may be due to the decreased activation of neurohormonal systems (the RAAS and the sympathetic nervous system) that occurs in patients with HFrEF taking long-acting diuretics, compared to short-acting loop diuretics [28].

Several studies in our review noted varying effects with increasing doses of loop diuretics. In their study of 2,338 patients with HFrEF who were taking loop diuretics, ter Maaten et al. found an increased independent risk of all-cause mortality and HF hospitalization with higher doses of loop diuretics [hazard ratio/doubling of loop diuretic dose: 1.06 (1.01-1.12), p=0.021] [30]. Ruocco et al. observed that the outcome event rate (death due to cardiovascular causes or HF hospitalization) was significantly higher (75% vs. 22%, p<0.001) in patients with HF with reduced ejection fraction (<50%) in the high-dose intravenous furosemide group compared to those receiving a low dose [31]. Onitsuka et al. reported that the use of high-dose furosemide (≥40 mg per day) was associated with an increased risk of cardiac events (cardiac death or re-hospitalization due to the worsening of HF) among HF patients with reduced ejection fraction (<45%) (adjusted HR=3.531, 95% CI=1.522-8.196, p=0.003) [32]. The association of poorer outcomes with higher doses of loop diuretics could be due to the worse baseline clinical state of the patients in the high-dose furosemide groups. These patients had higher New York Heart Association functional class, elevated brain natriuretic peptide, and blood urea nitrogen values at the time of admission [30-32].

On the contrary, He et al. did not observe any significant difference in mortality between the high- and low-dose loop diuretic patients with HFrEF. However, they reported that the incidence rates of hospitalization due to HF (p=0.045) were significantly lower in the high-dose group compared to the low-dose group of patients with HFrEF [33]. They also observed a significantly lower risk of composite clinical outcomes of death, total hospitalizations, and unscheduled visits due to HF among patients with HFrEF on high-dose furosemide compared to the low-dose group (p=0.01) [33].

Clinical Signs and Symptoms Resolution

He et al. observed significantly more weight loss at 72 hours among the high-dose furosemide group of patients with HFrEF than the low-dose group (weight change=-5.79 ± 10.68 lb in low-dose therapy vs. -9.17 ± 8.07 lb in the high-dose group; treatment difference=-3.30, 95% CI=-6.09 to -0.52 lb; p=0.02) [33]. They also reported a significantly higher number of patients free from congestion at 72 hours in the high-dose group (OR=2.36, 95% CI=1.06-5.24, p=0.04) [33]. This is expected as aggressive diuresis will help promote fluid removal, reducing fluid overload symptoms in patients with HFrEF. On the other hand, Ruocco et al. observed that diuretic response (daily weight loss per 40 mg of furosemide) at day three (0.106 (0.053-0.213) vs. 0.222 (0.127-0.407), p<0.001) and diuretic response during the entire infusion period (0.106 (0.064-0.240) vs. 0.266 (0.200-0.400), p<0.001) were lower in the high-dose intravenous loop diuretic group compared to the low-dose group, but no significant difference was observed at day one of admission [31]. They observed that patients who received high-dose intravenous loop diuretics had a significantly higher rate of congestion score >2 at the time of admission compared to the low-dose group (63% vs. 36%, p=0.003), so it is likely that this group of patients had more advanced disease and probably needed additional diuretic treatment that would stress the nephrons at a different site in order to achieve more efficient diuresis even with use of high-dose loop diuretics [31].

Efficacy of loop diuretics in HFpEF

All-Cause Mortality and HF Re-hospitalization

Mentz et al. reported no significant difference in all-cause mortality among patients with HF with EF ≥50% taking furosemide compared to torsemide for 12 months post-hospitalization (HR=0.88, 95% CI=0.66-1.17) [27]. In the same vein, Imaeda et al. reported no significant association between composite outcome (all-cause mortality and readmission rates) and use of short-acting or long-acting loop diuretics among patients with HFpEF (HR=0.9, 95% CI=0.70-1.17, p=0.43) [28].

A retrospective cohort study conducted by Parajuli et al. showed that the use of loop diuretics post-hospitalization is associated with a decreased 30-day hospital readmission risk in patients with HFpEF, and no significant association was seen with other cardioprotective medications, such as ACEIs, ARBs, beta-blockers, and aldosterone receptor antagonists [34]. Sharma et al. compared a continuous infusion diuretic strategy versus an intermittent bolus diuretic strategy among patients with HFpEF and reported no significant association between either strategy and several efficacy parameters, such as all-cause mortality at one year (p=0.34), 30-day HF readmission rate (p=0.35), and change in six-min walk distance at 72 h (p=0.77) [35]. HFpEF is commonly seen in younger patients with comorbidities such as hypertension and obesity [35]. Loop diuretics’ associated reduced readmission risk in patients with HFpEF may be because they reduce both blood pressure and help relieve congestion.

Safety of loop diuretics in HFrEF

Poor Renal Function

ter Maaten et al. reported that higher doses of loop diuretics are independently associated with an increased risk of worsening renal function in patients with HFrEF even after adjustment for propensity score and was independent of baseline serum creatinine values (odds ratio per doubling of loop diuretic dosage=1.33 (1.15-1.55), p<0.001) [30]. On the contrary, He et al. did not observe an increased risk of worsening renal function, as defined by the change in serum creatinine and cystatin C at 72 hours, among patients with HFrEF in the high-dose furosemide group compared to the low-dose group (serum creatinine, treatment difference=-0.05, 95% CI=-0.14 to 0.03 mg/dL, p=0.23; cystatin C, treatment difference=-0.06, 95% CI=-0.15 to 0.02 mg/dL, p=0.15) [33]. The detrimental effect of loop diuretics to worsen renal function is, perhaps, directly related to their reduction of renal blood flow through the tubuloglomerular feedback mechanism [30]. Higher doses of loop diuretics cause increased natriuresis and diuresis, which activates the tubuloglomerular feedback, resulting in afferent vasoconstriction and reduced renal blood flow [36]. ter Maaten et al. followed up the patients in their study for 12 months, whereas He et al. only observed safety outcomes for 72 hours. This could be why their study did not find a link between high-dose furosemide and deterioration of renal function.

Low Blood Pressure

ter Maaten et al. reported significantly lower blood pressures in the high-dose furosemide group compared to the low-dose group (p trend=0.031) [30]. Loop diuretics cause fluid and sodium loss. Hence, overzealous diuresis may reduce intravascular fluid volume and blood pressure.

Safety of loop diuretics in HFpEF

Sharma et al. observed that continuous infusion of furosemide was associated with a greater risk of worsening renal function compared to an intermittent bolus strategy among patients with HFpEF (OR=4.32, 95% CI=1.26-14.74, p=0.02) [35]. He et al. reported a significant increase in serum creatinine and cystatin C change at 72 hours among patients with HFpEF receiving high-dose furosemide compared to low dose (creatinine: treatment difference=0.16 mg/dL, 95% CI=0.02-0.30 mg/dL, p=0.03; cystatin C: treatment difference=0.26 mg/dL, 95% CI=0.09-0.43 mg/dL, p=0.003) [33]. This may be due to a fall in the already inadequate preload in HFpEF. Higher doses of loop diuretics will cause a significant reduction in intravascular volume and renal blood flow. A continuous infusion strategy may also not allow for adequate re-equilibration of intravascular and extravascular volumes in the setting of congestion with ongoing diuresis [35].

Limitations

This study has some limitations. Firstly, we searched only four databases and included only articles written in English and published after 2018. This may have excluded some articles with pertinent data on loop diuretic safety and efficacy in HF. The articles selected for this review also used different ejection fraction cutoffs for HF with reduced and preserved ejection fractions. Therefore, this limits the accurate comparability of the efficacy and safety outcomes of loop diuretics between the studies.

## Conclusions

Loop diuretics are efficacious for symptomatic relief in patients with HF. The choice of loop diuretics did not appear to impact clinical outcomes, such as mortality and rehospitalization, in patients with HFpEF or HFrEF despite differences in oral bioavailability and half-life. In clinical practice, patients with severe heart failure exacerbation are often treated with high doses of loop diuretics. Due to conflicting data, the impact of high versus low doses of loop diuretic therapy on clinical outcomes is a topic of debate. However, most studies suggest that higher doses of loop diuretics can lead to adverse clinical outcomes. Many studies have shown that a higher dose of loop diuretics can cause a decline in renal function and hypotension in those with HF, regardless of the type of HF. Due to the unclear effects of loop diuretics on clinical outcomes beyond symptomatic relief and poor safety outcomes associated with loop diuretics, discontinuation or reduction of dose should be considered in patients with HF after decongestion.
